# Comparative Analysis of Clinical, Hormonal and Morphological Studies in Patients with Neuroendocrine ACTH-Producing Tumours

**DOI:** 10.1155/2013/659232

**Published:** 2013-02-19

**Authors:** G. S. Kolesnikova, A. M. Lapshina, I. A. Voronkova, E. I. Marova, S. D. Arapova, N. P. Goncharov, I. I. Dedov

**Affiliations:** Endocrinological Research Center, Moscow 117036, Russia

## Abstract

This paper highlights the problem of neuroendocrine tumours (NETs) with clinical symptoms of hypercorticism caused by hypersecretion of adrenocorticotropic hormone (ACTH) by tumour cells. In most cases (85%), the tumours were localized in the pituitary gland (Cushing's disease); 15% of the patients had an extrapituitary tumour that manifest as an ectopic ACTH secretion (EAS). Comparative analysis of clinical, hormonal, histological, and immunohistochemical characteristics of pituitary and extrapituitary ACTH-secreting NET was performed. It included 46 patients with CD and 38 ones exhibiting ectopic ACTH secretion (EAS). Results of the study suggest differences between CD and EAS in terms of the severity of clinical manifestations and duration of the disease. Hormonal studies showed that EAS unlike CD was associated with high plasma ACTH and cortisol levels, late-evening salivary cortisol and daily urinary free cortisol, the absence of a 60% or greater reduction of cortisol in the HDDST test, and the presence of a low (less than 2) ACTH gradient in response to desmopressin administration with catheterization of cavernous sinuses. The study of morphofunctional characteristics of the removed NET demonstrated the ability of both pituitary and extrapituitary NETs to express ACTH as well as GH, PRL, LH, and FSH. The angiogenic markers (CD31 and VEGF) were detected with equal frequency regardless of the NET localization. The histological structure of all corticotropinomas suggested their benign origin, but extrapituitary NETs were represented by different morphological types with varying malignancy, invasiveness, and metastatic properties. A higher cell proliferation potential (Ki-67) was documented for NET in patients presenting with an ectopic ACTH secretion compared to those having corticotropinomas.

## 1. Introduction

NETs comprise a heterogeneous group of neoplasms originating from the neuroendocrine system. “Functioning” NETs that cause well apparent clinical symptoms due to hyperactivity of the involved hormones holds a special place in this group. CRH and ACTH-secreting tumours produce the clinical picture of hypercorticism. These tumours having a common origin in the cells of the diffuse neuroendocrine system, comparative analysis of the results of clinical, hormonal, histological, and immunohistochemical studies may contribute to the improvement of diagnostics of the disease and prognosis of remission after surgical intervention. 

## 2. Materials and Methods

A total of 84 patients were presenting with clinically manifest of hypercorticism were enrolled in the study. Diagnosis was made based on the results of hormonal studies, functional tests, brain MRI, MCTC imaging of adrenals, lungs, and mediastinum, kidneys and appendix. Blood samples for hormonal analysis were taken at 8.00 and 23.00 to elucidate circadian rhythms. ACTH and cortisol levels in peripheral blood, late-night free salivary cortisol, and 24-hr urinary free cortisol were measured by electrochemiluminescence immunoassay using an automated Cobas 6-1 system (Roche).

The patients were divided into two groups depending on NET localization. Group 1 was composed of 46 patients with pituitary NET (Cushing's disease, CD), and group 2 comprised 38 patients with extrapituitary NET (EAS). Group 1 consisted of 5 men and 41 women aged from 20 to 58 mean 39.5 +/− 10.2 years; duration of the disease 6.0 +/− 1.3 years. Group 2 included 14 men and 24 women aged from 21 to 70 mean 42 +/− 13 years; duration 2.89 +/− 1.5 years.

Forty practically healthy subjects (20 men and 20 women) at the age of 29.5 ± 11.2 years served as controls.

The removed tumours were fixed in a 10% neutral buffered formalin solution for 24 hours, dehydrated, compacted, and embedded in paraffin. Paraffin sections (5 mcm thick) were deparaffinized and stained with hematoxylin and eosin. Light microscopy was used to verify diagnosis of pituitary adenomas (basophilic, oxyphilic, chromophobic and mixed), carcinoid tumours (typical, atypical) of lungs, bronchi, kidneys, thymus, and small-cell lung carcinoma in accordance with the 1993 WHO histological classification of tumours (for pituitary adenomas), 2004 WHO classification (for ectopic ACTH-secreting lung and thymus tumours), and 2010 WHO classification (for ectopic ACTH-secreting appendix tumours). 

Forty six corticotropinomas and 30 ectopic ACTH-secreting tumours were studied immunohistochemically with the use of primary antibodies against ACTH, CRH, PRL, GH, LH, FSH, Ki-67, CD31, and VEGF. A sensitive complex of universal antibodies with biotin-free avidin-streptavidin-peroxidase system (Nichirei, Japan) was used as secondary antibodies and visualization system.

Tissues of 6 corticotropinomas and 3 tumours associated with ectopic ACTH secretion were examined using immunohistochemical double staining to identify two hormones at a time in a single section. For this purpose, an EnVision (Dako) G/2 Doublestain System, Rabbit/Mouse (DAB+/Permanent Red) was used.

Expression of ACTH, CRH, PRL, GH, LH, and FSH was assessed in 10 fields of view at ×400 as positive (staining of more than 10% tumour cells) or negative (staining of less than 10% cells). VEGF and CD31 expressions (10 fields of view, ×400) were described as positive and negative if more and fewer than 30% of the tumour cells were stained, respectively. Ki-67 labeling index (5 fields of view, ×400) was expressed as percentage of positively stained nuclei out of their total number in the tumour (*n* = 1000). 

The tissue of thyroid papillary cancer was used as positive control for CD31 and VEGF and the lymph node tissue for Ki-67.

### 2.1. Statistical Analysis

They were treated using Statsoft Statistica 8.0 for Windows, version 6. The normality of distribution was assessed by Shapiro-Wilk test. The data are presented as median ± SD. The Mann*-*Whitney test was used to calculate the statistical significance of intergroup differences. Analysis of qualitative characteristics in two independent groups was made using *χ* ^2^ criterion. The differences were considered statistically significant at *P* < 0.05.

## 3. Results 

All 84 patients were were presented with symptoms and clinical features of hypercorticism, such as central obesity, trophic skin lesions, arterial hypertension, cardiomyopathy, disturbed carbohydrate metabolism, secondary immunodeficiency, secondary hypogonadism, and systemic osteoporosis. Some differences in clinical symptoms were revealed between the groups (Table 1).

It was shown that 3 patients with EAS (group 2) unlike those with CD (group 1) exhibited cyclic symptoms of hypercorticism and their fast worsening, frequent hypokalemia (70% of the cases), and skin hyperpigmentation (92%). Moreover, 4 patients of group 2 suffered metastatic lesions in the adjacent organs absent in group 1. 

**Figure 1:**
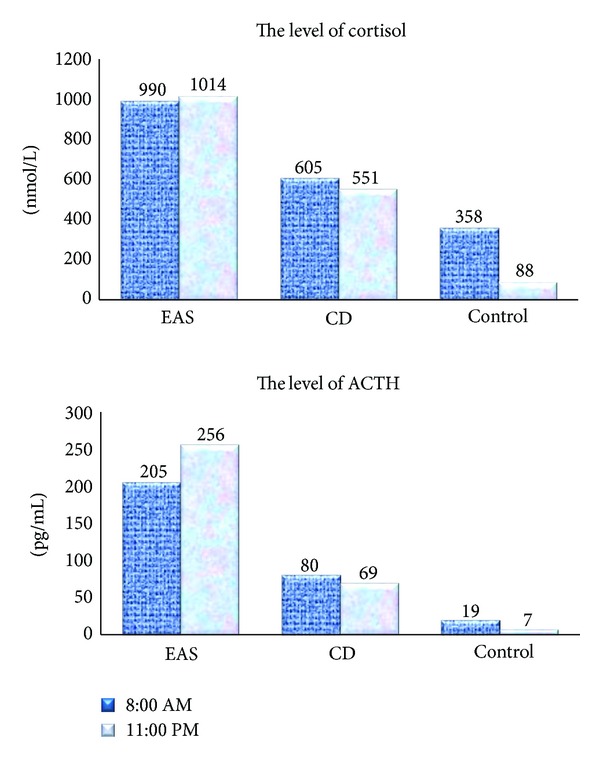
Cortisol (pmol/L) and ACTH (pg/mL) levels during a 24-hour period in patients with Cushing's disease and ectopic ACTH secretion compared with controls.

**Figure 2:**
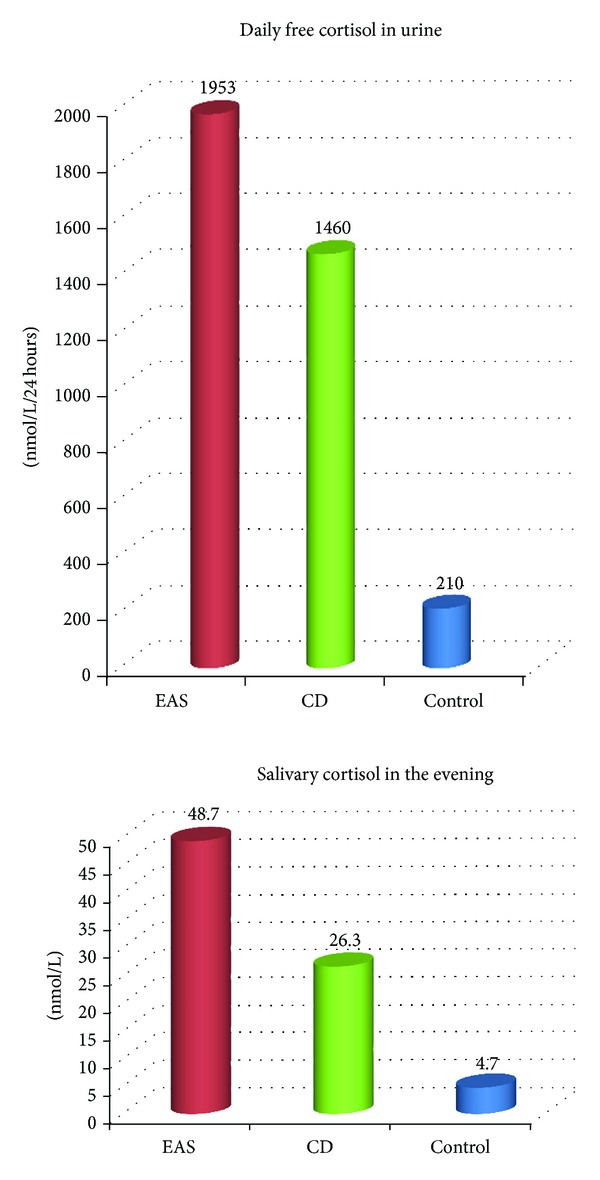
Free cortisol levels in 24-hr urine and saliva (measured at 23.00) in patients with Cushing's disease and ectopic ACTH secretion compared with controls.

In our study, plasma ACTH and cortisol, salivary cortisol, and 24-hr urinary free cortisol levels were significantly elevated in the patients of both groups (Figure 1). Also, plasma ACTH (*P* = 0.02) and cortisol (*P* = 0.0003) levels as well as daily urinary free cortisol (*P* = 0.03) in group 2 were significantly higher than in group 1. Moreover, the two groups were different in the degree of preservation of cortisol and ACTH circadian rhythms; specifically they were lost in the patients with EAS but persisted in group 2 even if they were less pronounced than in healthy subjects. Salivary cortisol levels measured at 23.00 were not significantly different between the groups but remained higher than in controls (*P* = 0.2) (Figure 2).

The dexamethasone suppression test (1 mg) yielded negative results in all the patients. The high-dose dexamethasone suppression test (HDDST, 8 mg) revealed differences in the regulation of the hypothalamo-pituitary-adrenal axis between the two groups. Specifically, 78% of the patients in group 1 experienced a 60% or greater decrease in the plasma cortisol level after dexamethasone administration. No similar drop was observed in group 2. It is concluded that HDDST is unsuitable for reliable differential diagnostics between CD and EAS.

Many authors emphasized difficulties of differential diagnostics between Cushing's disease and ectopic ACTH secretion [1–4]. Selective blood sampling from inferior petrosal sinuses after CRH or desmopressin stimulation has recently been described as the most informative method for differential diagnostics of ectopic ACTH secretion and Cushing's disease by virtue of its high sensitivity and specificity [5–8].

**Figure 3:**
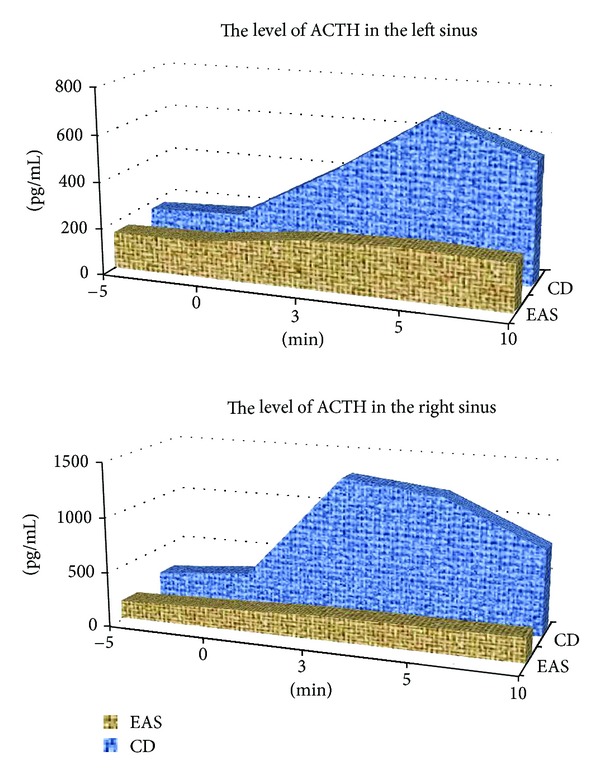
Dynamics of ACTH concentration based on selective blood sampling from petrosal sinuses in the desmopressin stimulation test in patients with Cushing's disease and ectopic ACTH secretion.

We obtained blood samples from inferior petrosal sinuses of 46 patients for differential diagnostics between CD and EAS in the desmopressin stimulation test using the generally accepted criteria to evaluate the results [5, 6]. The data obtained with catheterization of cavernous sinuses are presented in Table 2 and Figure 3.

Regarding Table 2 and Figure 3, the ACTH secretion was significantly increased in 31 patients with Cushing's disease, with ACTH gradient being above 2 under baseline conditions and above 3 after desmopressin stimulation. The maximum ACTH gradient in 15 patients of group 2 under basal and desmopressin stimulation conditions was below 2 which gave reason to suspect EAS. Their additional examination by adrenal, pulmonary, mediastinal, abdominal, retroabdominal, pelvic MSCT, gastroscopy, and colonoscopy revealed NET of different localization. All these tumours were surgically removed, and the diagnosis was confirmed by histological and immunohistochemical studies.

**Figure 4:**
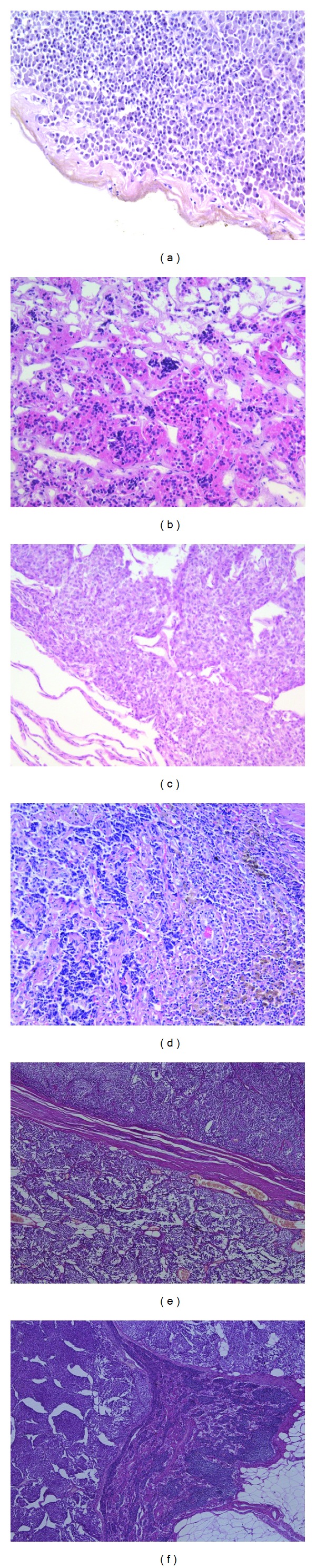
Histological structure of corticotropinomas and ectopic ACTH-secreting tumours (hematoxylin and eosin stainings). (a) A fragment of basophilic corticotropinoma, ×20. (b) Oxyphilic cell hyperplasia in adenohypophysis, ×20. (c) Typical lung carcinoid, ×20. (d) Metastasis of atypical lung carcinoid to a mediastinal lymph node, ×10. (e) and (f) Appendiceal NET (G2), ×10, 25 mm in diameter, clinically manifested as cyclic Cushing's syndrome with carcinoid crisis attacks between hypercortisolism cycles. (e) Invasion of the tumour into mesenteric fat. (f) NET metastasis to a mesenteric lymph node.

Histological data indicate that Cushing's disease in all cases was due to benign pituitary tumours (Figure 4). Analysis of tinctorial characteristics of 46 corticotropinomas demonstrated the predominance of basophilic adenomas (*n* = 24, 52.2%) followed by mixed-cell tumours (*n* = 19, 41.3%), chromophobic adenomas (*n* = 2, 4.3%), and oxyphilic adenoma (*n* = 1, 2.2%). Disturbed circulation was responsible for the formation of necrotic foci in 26 (56.5%) corticotropinomas, hemorrhagic areas, and stromal oedema in 5 (32.6%) and 11 (23.9%) tumours, respectively. Seventeen adenomas exhibited all three features; 4 adenomas contained necrotic foci and areas of hemorrhage, and 1 adenoma underwent necrosis and oedema. Tumour progression was associated with active neoplastic angiogenesis morphologically manifest as angiomatosis documented in 6 (13.0%) ACTH-secreting pituitary tumours. In 8 (17.4%) cases, pituitary adenoma coexisted with fragments of adenohypophyseal hyperplasia; four of them were mixed-cell hyperplasias (3 basophilic-oxyphilic, 1 oxyphilic-chromophobic) and the rest oxyphilic. 

Thus, all corticotropinomas were benign neoplasms dominated by basophilic cell tumours in terms of tinctorial characteristics (52.2%). These neoplasms underwent secondary changes, such as necrosis, hemorrhage, and stromal oedema, besides angiomatosis and mitoses. 

Postoperative material from 30 patients with ectopic ACTH secretion was available for morphological examination. Of these, 24 tumours were found in the bronchopulmonary system: 23 were lung carcinoids and 1 small-cell lung cancer. Four thymic tumours were represented by two atypical carcinoids, one typical carcinoid and one large-cell carcinoma. One tumour was typical renal carcinoid and one appendix neuroendocrine tumour (G2) (Figure 4). At the time of detection of the source of ACTH hypersecretion, four patients had metastatic lesions: in regional lymph nodes associated with 1 small-cell lung cancer, in 1 patient they were due to the extension of thymic AC onto the phrenic nerve, and in another case resulted from large-cell thymus NET spreading to the left brachiocephalic vein. Appendiceal NET (G2) extended to lymph nodes at aortic bifurcation and invaded into the cecal wall. Four patients suffered a relapse of cancer after surgical treatment. Two of them had lung carcinoids (with liver metastases in one case). One thymic TC produced metastases into neck lymph nodes, mediastinal pleura, and pelvic bones, and one AC gave metastases into paratracheal fat. Time to relapse varied from 1 to 5 years after removal of the primary tumour.

The study revealed ectopic ACTH-secreting tumours of different localization, morphological structure, and degree of malignancy (adenocarcinomas, thymic carcinomas, and small-cell lung cancer). Atypical lung carcinoids predominated. Metastases into various structures were documented in 4 cases. In contrast, all corticotropinomas were benign neoplasms with no signs of metastases or invasive growth. 

### 3.1. Immunoexpression of Pituitary Tropic Hormones in Pituitary and Extrapituitary ACTH-Secreting Tumours

**Figure 5:**
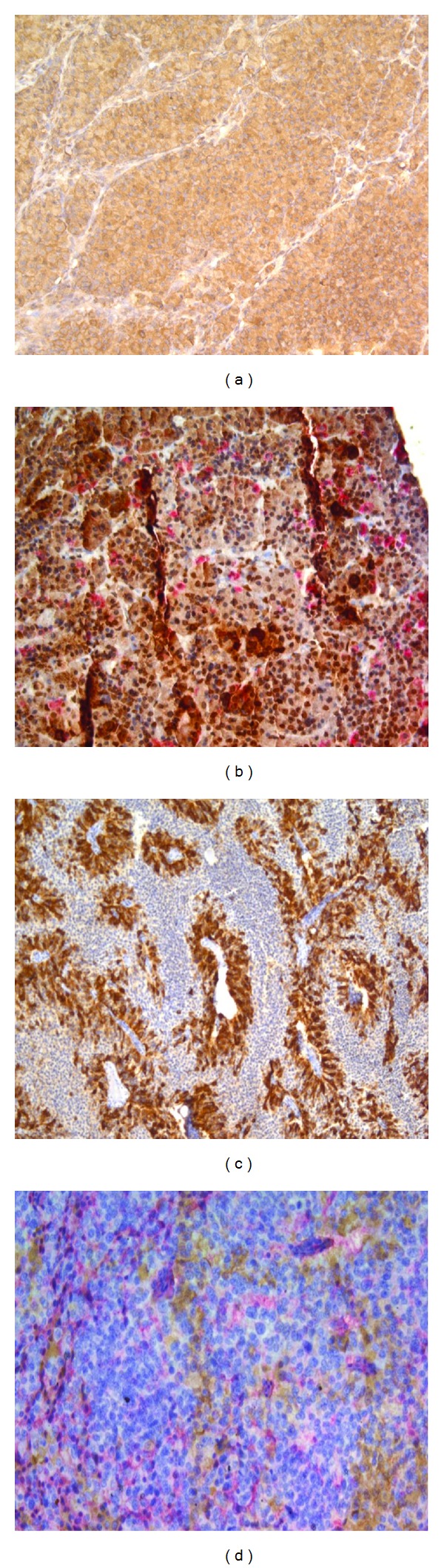
Expression of pituitary tropic hormones in corticotropinomas and ectopic ACTH-secreting tumours. (a) Indirect immunoperoxidase staining of corticotropinoma cells with anti-ACTH antibodies, ×20. (b) Indirect double immunoperoxidase staining of corticotropinoma cells with anti-ACTH and anti-LH antibodies, ×20. (c) Indirect immunoperoxidase staining of atypical lung carcinoid cells with anti-ACTH antibodies, ×10. (d) Indirect double immunoperoxidase staining of atypical carcinoid cells with anti-ACTH and anti-CRH antibodies, ×20.

Immunohistochemical reactions with antibodies against ACTH, PRL, GH, LH, and FSH were performed for comprehensive functional assessment in 46 corticotropinomas and 18 ACTH ectopic tumours (all EAS neoplasms were examined for CRH expression, none of them expressed FSH). Cytoplasmic expression of all hormones was measured (Figure 5). Staining density of cytoplasm stained with antibodies to GH, PRL, and LH was lower than that of cytoplasm stained with anti-ACTH and anti-CRH antibodies. The distribution of corticotropinomas and ACTH ectopic tumours by hormone expression is illustrated by Table 3. 

Six bi- and polyhormonal corticotropinomas were double labeled with antibodies against pituitary tropic hormones using an immunohistochemical method. The same technique was employed to stain 3 ectopic ACTH-secreting tumours with anti-ACTH and anti-CRH antibodies. This procedure was performed to confirm expression of two hormones in a single tumour section and to identify hormone-synthesizing cells (Figures 5(b) and 5(d)). Cytoplasm of ACTH-secreting cells stained brown, that of the cells synthesizing other hormones (GH, PRL, LH, FSH, and CRH) stained pink. This immunohistochemical procedure confirmed the results obtained with the use of a single type of antibodies and revealed cells expressing different hormones in one section of a given tumour. 

### 3.2. Immunoexpression of the Proliferative Activity (Ki-67) and Angiogenesis (CD31 and VEGF) Markers in Pituitary and Extrapituitary ACTH-Secreting Tumours

**Figure 6:**
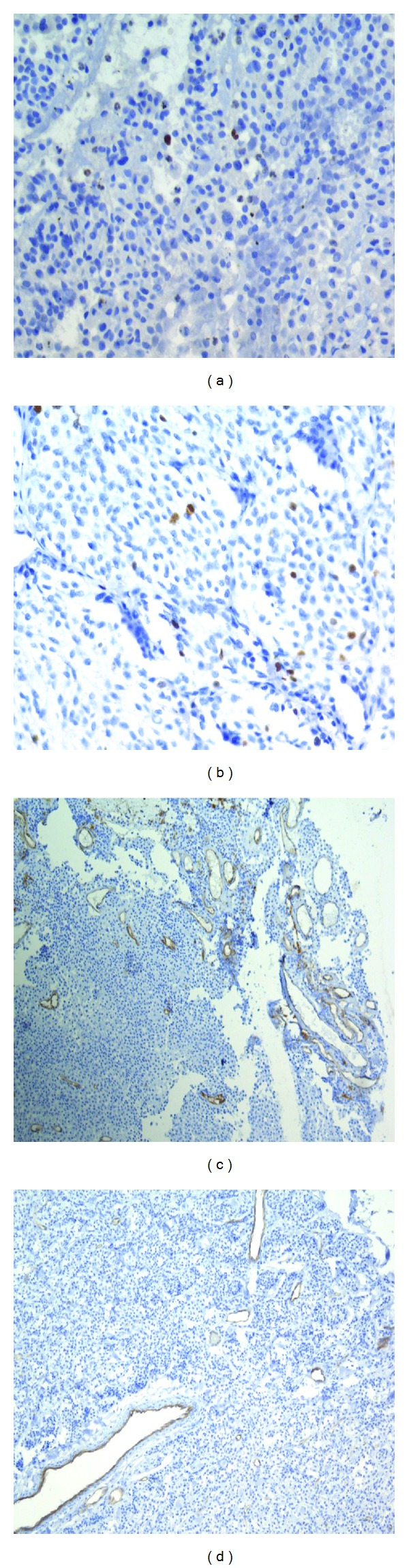
Expression of Ki-67, CD31 in corticotropinomas and ectopic ACTH-secreting tumours. (a) Indirect anti-Ki-67 immunoperoxidase staining of corticotropinoma cell nuclei, ×40. (b) Indirect anti-Ki-67 immunoperoxidase staining of the cell nuclei in atypical lung carcinoid, ×40. (c) Indirect anti-CD31 immunoperoxidase staining of vascular endothelium in corticotropinomas, ×10. (d) Indirect anti-CD31 immunoperoxidase staining of vascular endothelium in typical lung carcinoid, ×10.

Immunohistochemical staining revealed the expression of Ki-67 in tumour cell nuclei (Figures 6(a) and 6(b)), CD31 in endothelial cells of the tumour, and vascular endothelial growth factor (VEGF) in tumour cell cytoplasm (Figures 6(c) and 6(d)). The immunohistochemical procedure using the above antibodies was performed to stain 46 corticotropinomas. Anti-Ki-67 antibodies were used to examine 20 EAS tumours and antibodies to CD31 and VEGF to stain 14 such tumours. 

Expression of Ki-67 was demonstrated in 45 (97.8%) corticotropinomas. The labeling index of this marker varied from 0.07% to 0.46% (median 0.14%). Ki-67 expression was detected in all ectopic tumours, with a median labeling index of 1.3% (maximum 2.5%, minimum 0.5%) for typical carcinoids (*n* = 5), 3.87% (maximum 16.4%, minimum 0.32%) for atypical carcinoids (*n* = 13), 25% for a large-cell NE carcinoma, and 18.5% for a NE appendiceal tumour (G2). Ki-67 labeling indices in corticotropinomas and ectopic tumours were significantly different (*P* < 0.0000001). 

Expression of the angiogenesis marker CD31 was documented in 24 corticotropinomas (52.2%) and VEGF in 28 adenomas (60.9%). CD31 was detected in EAS tumours: one SCLC, 5 AC (two metastatic), and 2 TC. VEGF was present in 2 AC (without metastases) and 1 TC. Statistical analysis of CD31 (*P* = 0.3) and VEGF (*P* = 0.7) expression rates in corticotropinomas and ectopic tumours did not reveal significant differences. 

Thus, immunohistochemical studies showed that ACTH-expressing monohormonal tumours (69.6%) prevailed among the corticotropinomas, the rest of them (30.4%) expressed other pituitary tropic hormones, besides ACTH determining the clinical presentation of the disease. EAS tumours (33.3%) were shown to synthesize either ACTH or CRH responsible for the development of hypercorticism or coexpress ACTH and CRH (*n* = 5; 27.8%). Ectopic mono-, bi-, and polyhormonal tumours occurred equally often (33.3%). Corticotropinomas and ectopic ACTH-secreting tumours were not significantly different in terms of production of one (*P* = 0.2), two (*P* = 0.1), and more hormones (*P* = 0.2). Ki-67 labeling index in EAS tumours was significantly higher than in corticotropinomas. 

Most corticotropinomas and ectopic ACTH-secreting tumours expressed CD31 and VEGF. There was no significant difference in the expression rate of these markers between the two types of tumours.

## 4. Discussion 

This comparative study demonstrated differences in the clinical presentation of Cushing's disease and ectopic ACTH secretion. For example, ectopic ACTH secretion is more frequently associated with hypokalemia (73%), melanoderma (92.9%), and pronounced myopathic syndrome (42.9%). These findings agree with the literature data [9, 10]. Moreover, the time span between the first symptoms of the disease and its definitive diagnosis is longer in the patients presenting with CD than in those with ectopic ACTH secretion. Hormone analysis shows that ACTH and cortisol levels are elevated in both ectopic ACTH secretion and Cushing's disease, but the magnitude of elevation differs.

For example, blood ACTH level in patients with ectopic ACTH secretion was significantly higher than in Cushing's disease. The daily rhythm of cortisol and ACTH was preserved differently in the two groups. It was virtually absent in patients with ectopic ACTH secretion but persisted in Cushing's disease even if it was less apparent than in the control group. Probably, the hypothalamo-pituitary axis of some CD patients remains sensitive to hypothalamic and suprahypothalamic regulators of circadian rhythms of these hormones.

Many authors emphasize difficulties encountered in differential diagnostics of ectopic ACTH secretion and Cushing's disease. Selective blood sampling from inferior petrosal sinuses after CRH or desmopressin stimulation has recently been proposed as the most reliable method for differential diagnostics of ectopic ACTH secretion and Cushing's disease [6–9]. Our study confirmed its high sensitivity (100%) and specificity (100%). 

In CD patients, all tumours had a structure of pituitary adenomas. Eight cases (17.4%) had in addition fragments of adenohypophyseal hyperplasia. EAS tumours included carcinoids affecting different organs and SCLC characterized by higher malignant potential compared with corticotropinomas; they showed an infiltrative growth pattern and caused metastatic lesions in various tissues.

Immunohistochemical studies using antibodies against pituitary tropic hormones showed that the number of ACTH and CRH-secreting cells in pituitary and extrapituitary tumours is much greater than the number of cells expressing other hormones. Anti-ACTH and anti-CRF antibodies staining density was higher than in the studies with antibodies against GH, PRL, LH, and FSH. These findings are in agreement with those reported by other authors [11, 12]. GH, PRL, LH, and FSH levels in patients with CD and EAS are usually not very high due to the small number of cells secreting these hormones or due to the production of defective molecules rapidly decaying and unable to enter the systemic circulation [13–15]. We observed only 3 cases of isolated CRH expression in an ectopic source (without ACTH synthesis) that might be responsible for corticotroph hyperplasia and excess pituitary ACTH production [16]. Coexpression of ACTH and CRH was documented in 5 cases. There was no significant difference between expression patterns of mono- (*P* = 0.9), bi- (*P* = 0.9), and polyhormonal (*P* = 0.7) corticotropinomas and EAS tumours. The ability to express similar hormones by such structurally different neoplasms is attributable to their common origin [17, 18]. 

We estimated the malignancy potential of ACTH-secreting pituitary tumours and extrapituitary NE neoplasms by studying expression of factors controlling cell proliferation (Ki-67) and angiogenesis (CD31 and VEGF).

We documented expression of Ki-67 by dividing cells in all phases of the cell cycle; it was present in the cell nuclei of practically all tumours excepting one corticotropinomas. Analysis of Ki-67 labeling indices in corticotropinomas and ectopic tumours revealed significant difference in the expression of this marker (*P* < 0.000001). Enhanced expression of Ki-67 suggests more active proliferation in EAS tumours [11]. 

Neoangiogenesis in a tumour is an important precondition for neoplasm growth and spread due to activation of proangiogenic growth factors (with VEGF being the key one), suppression of inhibitors of angiogenesis (angiostatin, thrombospondins, etc.), and interaction of these factors with tumour stroma. Metalloproteinases (collagenases) produced in the stroma destroy its structure thereby promoting formation of new vessels and tumour progression. Activity of metalloproteinase inhibitors decreases. CD31 is an adhesion molecule of epithelial cells and platelets that can be used to label tumour vascular endothelium and serves as an indicator of angiogenic activity. Our study revealed positive expression of CD31 in 24 (52.2%) corticotropinomas, SCLC, and 4 atypical carcinoids (with mediastinal lymph node metastasis in SCLC and two AC) that reflected the angiogenic activity of the tumours. Turner et al. [19, 20] reported a significantly lower vascular density based on the study of CD31 expression in corticotropinomas. However, we did not found significant differences between CD31 expression rates in CD31 expression between corticotropinomas and ectopic tumours (*P* = 0.7). 

The intensity of neoangiogenesis is related to VEGF [21] expression and concentration. VEGF expression was observed in 28 pituitary adenomas and 3 ectopic tumours (2 AC and 1 TC). Immunohistochemical studies of neuroendocrine tumours usually discover VEGF in cell cytoplasm [12, 20]. There was no significant difference in the expression rate of this marker in corticotropinomas and ectopic tumours (*P* = 0.2), nor was it different between various ectopic tumours, such as typical and atypical carcinoids having metastatic potential, and neoplasms forming no metastases. These findings are confirmed by the study of  Turner et al. [20]. 

To sum up, the Ki-67 labeling index was the only marker of differentiation between malignant potentials of corticotropinomas and ectopic ACTH-secreting tumours in our study. These neoplasms had similar characteristics of cellular adhesion and angiogenesis regardless of their histological structure. 

## 5. Conclusion 

Comprehensive comparative analysis of clinical, hormonal, histological, and immunohistochemical studies of pituitary and extrapituitary ACTH-secreting NET revealed a number of similar and different features. The clinical picture of hypercorticism due to EAS tumours, unlike CD, is characterized by rapid deterioration of specific symptoms of the disease including marked hypokalemia, skin hyperpigmentation, high evening plasma and salivary ACTH and cortisol levels as well as daily urinary free cortisol, the absence of a 60% or greater reduction of cortisol in the HDDST test, and the presence of a low (less than 2) cortisol gradient in response to desmopressin administration with catheterization of cavernous sinuses.

Histological and immunohistochemical investigations of pituitary and extrapituitary ACTH-secreting NET also demonstrated similar and different features. Similarity between corticotropinomas and ectopic ACTH-secreting tumours included their ability to express not only ACTH (or CRH in EAS neoplasms), but also two or more pituitary tropic hormones (GH, PRL, LH, and FSH) and proangiogenic markers (CD31 and VEGF), with equal frequency. Pituitary and extrapituitary ACTH-secreting tumours differed in histological structure and malignant potential. All corticotropinomas were benign neoplasms, whereas ectopic tumours were different in terms of morphological structure and degree of malignancy (AC, TC, SCLC, and large-cell NE carcinoma); some of them possessed metastatic activity. Cellular proliferation (Ki-67 labeling index) was especially pronounced in ectopic ACTH-secreting tumours.

## Figures and Tables

**Table 1:** Frequency of main symptoms and complaints in patients with CD and EAS.

Complaints/symptoms	% of the total number of patients with CD	% of the total number of patients with EAS
Overweight	90.0	70.0
Underweight	10.0	30.0
Melanoderma	10.9	92.0
Myopathic syndrome	30.0	42.9
Purple striae	67.0	50.0
Skin impurity	10.9	16.7
Excessive facial hair growth	41.3	16.7
Hypokalemia	38.0	73.0
Arterial hypertension	82.6	72.2
Encephalopathy/depression	64.0	54.0
Amenorrhea in women	53.7	10.0
Reduced sexual potency in men	40.0	30.0
Back pain	57.0	38.9
Diabetes mellitus	40.0	38.0
Systemic osteoporosis	93.0	85.0
Nephrolithiasis/urolithiasis/chronic pyelonephritis	65.0	46.0

**Table 2:** Plasma ACTH gradient revealed by selective blood sampling from petrosal sinuses in the desmopressin stimulation test in patients with Cushing's disease and ectopic ACTH secretion.

ACTH gradient Center/periphery	Cushing's disease (*n* = 31)	Ectopic ACTH secretion (*n* = 15)
Baseline level	Above 2	Below 2
After stimulation	Above 3	Below 2
Intersinus gradient	Above 2.4	Below 1.4

**Table 3:** Distribution of pituitary and extrapituitary ACTH-secreting tumours based on the results of immunoexpression of tropic hormones.

Hormone expression	Corticotropinomas, *n* = 46	Ectopic ACTH-secreting tumours		*P*, *χ* ^2^
		IHC characteristics	Morphology	
Monohormonal	ACTH, *n* = 32 (69.6%)	Total, *n* = 6 (33.3%): ACTH (*n* = 4) CRH (*n* = 2)	3 AC/1 TC 1 AC/1 TC	0.2

Bihormonal	Total, *n* = 15 (32.6%): ACTH-PRL, *n* = 7 ACTH-FSH, *n* = 1 ACTH-STH, *n* = 4 ACTH-LH, *n* = 3	Total, *n* = 6 (33.3%): ACTH-CRH, *n* = 5 ACTH-PRL, *n* = 1	2 AC/2 TC/1 SCLC 1 TC	0.1

Polyhormonal	Total, *n* = 9 (19.6%): ACTH-STH-PRL, *n* = 4 ACTH-STH-PRL-FSH, *n* = 1 ACTH-STH-LH-FSH, *n* = 1 ACTH-PRL-LH-FSH, *n* = 1 ACTH-STH-PRL-LH-FSH, *n* = 1 ACTH-PRG-LH, *n* = 1	Total, *n* = 6 (33.3%): ACTH-CRH-STH, *n* = 2 ACTH-STH-PRL, *n* = 1 CRH-STH-PRL, *n* = 1 CRH-STH-PRL-LH, *n* = 1 ACTH-CRH-STH-PRL, *n* = 1	2 AC 1 AC 1 TC 1 TC 1 TC	0.2

## Abbreviations

ACTH: Adrenocorticotropic hormone
AC: Atypical carcinoid
EAS: Ectopic ACTH secretion
CD: Cushing's disease
IHCS: Immunohistochemical study
CRH: Corticotropin-releasing hormone
LH: Luteinizing hormone
SCLC: Small-cell lung cancer
NET: Neuroendocrine tumour
PRL: Prolactin
GH: Growth hormone
TC: Typical carcinoid
FSH: Follicle-stimulating hormone
Ki-67: Marker of proliferative activity
CD31: Angiogenic marker
VEGF: Vascular endothelial growthfactor.
